# Preliminary Experience of Transurethral Thulium Laser En Bloc Resection of Paraganglioma of the Urinary Bladder

**DOI:** 10.1245/s10434-025-18692-w

**Published:** 2026-01-25

**Authors:** Yongjun Yang, Ye Wu, Kehao Yang, Guangqing Song, Zhe Liu, Qiang Lu, Yuanwei Li

**Affiliations:** Department of Urology, Hunan Provincial People’s Hospital, The First Affiliated Hospital of Hunan Normal University, Changsha, Hunan Province People’s Republic of China

**Keywords:** Bladder, Paraganglioma, Thulium laser, En bloc resection, Catecholamine, Blood pressure fluctuations

## Abstract

**Background:**

The use of thulium laser en bloc resection (TmLER) for treating bladder tumor has gained increasing attention in recent years. This study aims to share our preliminary clinical experience with respect to the safety and efficacy of TmLER in treating paraganglioma of the urinary bladder (PUB).

**Patients and Methods:**

The clinical and pathological data of eight patients with PUB who underwent TmLER under general anesthesia at our urology center between January 2020 and April 2024 were retrospectively collected and analyzed. Detailed documentation was maintained regarding intraoperative blood pressure fluctuations, the incidence of perioperative complications, changes in postoperative catecholamine hormone levels, and tumor recurrence rates.

**Results:**

Eight patients successfully underwent TmLER without the need for conversion to conventional transurethral resection. The operation duration ranged from 25 to 37 min, with an average of 28.9 min. During TmLER, systolic blood pressure fluctuated between 8 and 20 mmHg, averaging 13.5 mmHg. No significant perioperative complications were observed. Postoperatively, bladder irrigation was discontinued on average after 18.75 h, and the urinary catheter was removed after 3–5 days. Hospital stays ranged from 4 to 6 days, with an average of 4.91 days. At the 1-month follow-up, there was no significant fluctuation in systolic blood pressure before and after urination, and plasma catecholamine hormone levels had normalized. Furthermore, no tumor recurrence was detected at the 12-month follow-up.

**Conclusions:**

For the treatment of PUB, the TmLER technique can effectively reduce intraoperative bleeding and blood pressure fluctuations, and it is a safe and efficient minimally invasive surgical option.

Pheochromocytoma is a neuroendocrine tumor arising from chromaffin cells, which can intermittently or continuously release excessive amounts of catecholamines, primarily norepinephrine, epinephrine, and dopamine.^1^ Approximately 80% of pheochromocytoma arise from the adrenal medulla, whereas the remaining 20% develop in extra-adrenal paraganglia. Extra-adrenal pheochromocytoma, also referred to as paraganglioma, arises from chromaffin tissue located outside the adrenal medulla within the sympathetic or parasympathetic nervous system.^2^ These tumors frequently occur in various locations, including the head and neck, retroperitoneal area, mediastinum, and bladder.^3,4^ Paraganglioma of the urinary bladder (PUB) is a rare neoplasm arising from paraganglionic cells located in the bladder wall. It represents approximately 10% of extra-adrenal pheochromocytoma, yet accounts for around 0.06% of all bladder tumors.^5^ Functional PUB is capable of secreting catecholamines, which may result in patients diagnosed with PUB experiencing a range of clinical symptoms, including headache, palpitations, and a significant rise in blood pressure during urination.^6^

The surgical management of PUB primarily encompasses transurethral resection of bladder tumor (TURBT), partial cystectomy, and radical cystectomy.^7^ Among these options, TURBT is widely recognized as a suitable treatment approach for isolated PUB lesions that are relatively small in diameter.^8^ Currently, the thulium laser, as an energy source, has gained widespread application in the surgical treatment of bladder tumor, particularly in transurethral en bloc resection.^9^ Clinical studies have demonstrated that thulium laser en bloc resection (TmLER) not only ensures superior safety, but also delivers excellent oncological outcomes.^10,11^ Owing to its shallow penetration depth, the thulium laser enables precise cutting of tumor tissues. Moreover, it exhibits remarkable hemostatic capabilities by effectively coagulating and blocking the blood vessels supplying the tumor, thereby significantly minimizing intraoperative bleeding. On the basis of these considerations, we implemented the TmLER technique for the treatment of PUB and present the preliminary outcomes of its clinical application.

## Patients and Methods

### Patients

From January 2020 to April 2024, we retrospectively collected data on patients who were pathologically diagnosed with PUB and underwent TmLER at our medical center. A total of eight patients were recruited for this study, consisting of three male and five female individuals. The patients’ age range spanned from 28 to 61 years, with a mean age of 43 years. The baseline systolic blood pressure range was reported to be between 109 and 130 mmHg, with a mean value of 121.25 mmHg. All patients experienced intermittent dizziness and palpitations following urination. Systolic blood pressure measurements taken within 5 min post-urination revealed an increase of 40 to 72 mmHg from baseline, with a mean elevation of 56.38 mmHg. Preoperative computed tomography urography (CTU) revealed a solitary space-occupying lesion within the bladder wall. Specifically, among the eight patients, three presented with tumors on the left lateral wall of the bladder, two on the right lateral wall, two on the posterior wall, and one on the anterior wall. The tumor sizes ranged from 1.2 to 2.7 cm in diameter, with an average diameter of 2.0 cm.

Preoperative plasma epinephrine concentrations ranged from 521.8 to 1038.3 pmol/L, with a mean of 775.4 pmol/L (reference range 0–605.4 pmol/L). Plasma dopamine concentrations varied between 175.6 and 561.2 pmol/L, averaging 335.9 pmol/L (reference range 0–195.7 pmol/L). Furthermore, the 24-h urinary vanillylmandelic acid excretion was measured at 8.2 to 16.3 mg/24 h, with a mean excretion of 13.6 mg/24 h (reference range 0–12 mg/24 h). Among the total of eight cases, a comprehensive analysis of medical history and clinical data indicate that PUB is the most probable diagnosis. Preoperatively, all patients were managed with oral prazosin hydrochloride for blood pressure control and received intravenous fluid resuscitation to address the issue of insufficient effective circulating blood volume.

### Surgical Procedure

The TmLER surgery, guided by white light cystoscopy, was successfully performed by an experienced senior urologist. As previously reported by our research team,^12^ all patients were positioned in the lithotomy position following the induction of general anesthesia to facilitate surgical intervention. During surgical procedure, continuous bladder irrigation was maintained via the cystoscopy’s water inlet using sterile normal saline to ensure optimal visualization of the surgical field. A thulium laser treatment system (manufactured by Shanghai Ruikeen Laser Technology Co., Ltd., Shanghai, China) served as the energy source, with the power set at 20 W. The cystoscopy was gently inserted through the urethra into the bladder. The urologist performed a thorough and detailed examination of the entire bladder cavity, meticulously documenting the precise location, diameter, and morphological feature of the tumor. A laser fiber was introduced through the working channel of the cystoscopy. The blood vessels supplying the tumor tissue were coagulated and blocked using laser energy. Subsequently, a circular mucosal incision was performed at a safe distance of 5–10 mm from the tumor margin. On the pre-marked circle, a precise vertical incision is made extending from the bladder mucosa layer to the deep muscular layer. Thereafter, by integrating the sharp cutting effect of laser energy with the blunt dissection capability of the cystoscope operating rod, the tumor tissue is progressively separated and completely enucleated at the deep muscular layer. To minimize cauterization artifacts at the tumor base, laser energy is mainly employed for precise dissection of muscle fibers surrounding the tumor. Subsequently, using the outer sheath of the cystoscopy, the intact tumor specimen is retrieved via the Ellick’s evacuator.

### Postoperative Management and Follow-Up

After the operation, it is essential to closely monitor the dynamic changes in the patient’s vital signs, with particular emphasis on key indicators such as blood pressure, pulse rate, and oxygen saturation. Concurrently, continuous low-flow irrigation of the bladder with normal saline should be performed, and intravenous administration of antispasmodic drug was initiated to effectively alleviate symptoms of bladder spasms. On the first postoperative morning, venous blood was collected from the patient in a fasting state for a routine blood test to compare and analyze the changes in hemoglobin levels before and after the surgery. The first follow-up was performed 1 month postoperatively. The follow-up primarily focused on assessing the alleviation of clinical symptoms, the presence or absence of hematuria, the occurrence of urinary difficulty, and the measurement of systolic blood pressure 5 min after urination. Within the first year post-surgery, patients are recommended to undergo cystoscopy at 3-month intervals and CTU every 6 months to monitor for potential tumor recurrence.

### Statistical Methods

The clinical pathological data of patients were analyzed using Graphpad Prism 9.0 statistical software. Continuous variables were expressed as average and range, and count data were presented as example (*n*) and percentage (%). The data before and after operation were compared using paired *t*-test, with *P* < 0.05 considered statistically significant.

## Results

All eight patients in this group underwent successful TmLER surgery. Operation times varied between 25 and 37 min, with a mean duration of 28.9 min. Hemoglobin levels changed from 1 to 8 g/L pre- to post-operation, with an average change of 3.75 g/L. Intraoperative systolic blood pressure fluctuated within a range of 8–20 mmHg, with an average variation of 13.5 mmHg. Notably, one patient exhibited a sudden increase in systolic blood pressure to 20 mmHg. This patient’s tumor was located on the anterior wall of the bladder. Following targeted symptomatic management, blood pressure successfully returned to baseline. The remaining seven patients did not experience any significant fluctuations in blood pressure. Furthermore, none of the eight patients encountered complications such as bladder perforation or obturator nerve reflex during the procedure, and no perioperative blood transfusions were required. On the basis of the modified Clavien–Dindo classification system for surgical complications,^13^ the TmLER procedure was conducted without any intraoperative complications reaching or exceeding grade II.

Continuous dynamic monitoring of vital signs in the ward revealed that none of the patients experienced adrenal crisis, abrupt blood pressure elevation, fever, or other complications post-surgery. Postoperatively, continuous bladder irrigation was maintained without the formation of blood clots or catheter obstruction being observed. Bladder irrigation was discontinued on the morning of the second postoperative day, with an average duration of 18.75 h. The indwelling catheter was subsequently removed between the third and fifth postoperative days, with an average dwell time of 3.91 days. Postoperative pathological examination confirmed the diagnosis of PUB, with negative tumor margins and an intact capsule.

**Fig. 1:**
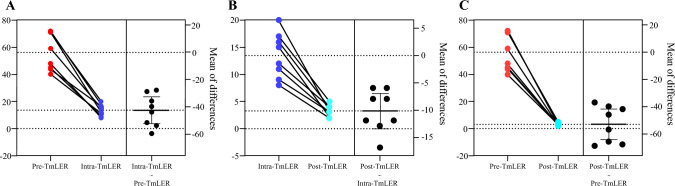
A comparative analysis of systolic blood pressure fluctuations in three different periods; **A** comparative analysis of systolic blood pressure fluctuations during preoperative urination and intraoperative tumor resection; **B** comparative analysis of systolic blood pressure fluctuations during intraoperative tumor resection and postoperative urination; **C** comparative analysis of systolic blood pressure fluctuations during preoperative urination and postoperative urination

The postoperative follow-up duration extended from 13 to 50 months, with a median follow-up time of 35.5 months. Among the eight patients evaluated 1 month after surgery, none reported dizziness during urination, nor were there any significant symptoms of dysuria, hematuria, or urinary tract irritation. Systolic blood pressure monitoring revealed fluctuations ranging from 2 to 5 mmHg before and after urination, with an average fluctuation of 3.25 mmHg. In Fig. 1, the systolic blood pressure fluctuations across three distinct periods were analyzed. The results indicated that both the tumor resection and postoperative urination phases exhibited significantly reduced blood pressure fluctuations compared with the preoperative urination phase. Additionally, a more detailed comparison demonstrated that the postoperative urination phase had markedly lower blood pressure fluctuations than the tumor resection phase (*P* < 0.001). At 12-month follow-up, cystoscopy and CTU confirmed no evidence of tumor recurrence.

## Discussion

PUB is a rare neuroendocrine tumor originating from paraganglionic cells in the bladder wall, representing less than 10% of all paraganglioma. This tumor can occur at any age but predominantly affects individuals aged 30–50 years, with a slightly higher prevalence in women compared with men.^14^ Depending on whether the tumor exhibits neuroendocrine functionality, PUB can be categorized into functional and nonfunctional subtypes. Approximately 50% of cases are functional PUB, which secrete varying amounts of catecholamines, thereby inducing clinical symptoms such as symptomatic hypertension, dizziness, and palpitations during urination due to catecholamine release by the tumor tissue.^15^ Conversely, nonfunctional PUB typically presents with hematuria or other nonspecific symptoms. Due to the absence of characteristic clinical features, preoperative misdiagnosis rates are relatively high, and definitive diagnosis often relies on postoperative pathological examination results.^16^ Given the intricacies associated with preoperative PUB diagnosis, imaging analysis and laboratory testing play crucial roles. On imaging studies, PUB frequently manifests as a hypervascular mass that demonstrates marked enhancement on computed tomography (CT) contrast scans and follows a “fast in, slow out” enhancement pattern. Moreover, patients with functional PUB exhibit elevated levels of catecholamines in both blood and 24-h urine samples, which serve as critical indicators for preoperative etiological diagnosis.

The treatment of PUB primarily focuses on surgical resection. According to systematic review analyses, 20% of patients undergo TURBT, 70% undergo partial cystectomy, and 10% undergo radical cystectomy.^17^ For larger PUB, complete transurethral resection can be technically challenging, leading clinicians to prefer partial or radical cystectomy in such cases.^18^ Currently, laparoscopic partial cystectomy has become a relatively mature technique, and tertiary hospitals routinely perform these procedures. However, during the surgical management of functional PUB, traction or cutting operations may stimulate the tumor to release large amounts of catecholamines instantaneously, potentially causing abrupt spikes in blood pressure. To address this issue, some scholars have suggested combining transurethral en bloc resection with laparoscopic surgery for treating functional PUB, which could reduce the incidence of intraoperative blood pressure fluctuations.^19^ For nonfunctional PUB, transurethral resection is a safe and effective treatment option.^20^ For functional PUB, under the premise of precise preoperative diagnosis and adequate preparation, transurethral resection shows similar performance in terms of surgical safety and postoperative recurrence risk compared with partial cystectomy.^8^ Compared with conventional TURBT, en bloc resection of bladder tumor (ERBT) avoids direct manipulation of tumor tissue, which may trigger the release of catecholamines, and thereby theoretically minimizes the risk of intraoperative sudden blood pressure elevation. For smaller PUB tumor, transurethral resection remains a safe and effective surgical option (Table 1).

**Table 1:** Clinical characteristics and perioperative data of the patients

	Results
Number (*n*)	8
Age (years)	20–61 (43)
Gender	
Male	3
Female	5
Tumor diameter (cm)	1.2–2.7 (2.0)
Tumor location, *n* (%)	
Lift wall	3 (37.5%)
Right wall	2 (25.0%)
Posterior wall	2 (25.0%)
Anterior wall	1 (12.5%)
Operative time (min)	25–37 (28.9)
Complications, *n* (%)	
Grade I	1 (12.5%)
Grade II	0 (0.0%)
Obturator nerve reflex, *n* (%)	0 (0.00%)
Bladder perforation, *n* (%)	0 (0.00%)
Hemoglobin loss	1–8 (3.8)
Irrigation (h)	16–22 (18.8)
Catheterization (day)	3.7–4.7 (3.9)
Recurrence, *n* (%)	0 (0.00%)

In recent years, ERBT has gradually garnered significant attention in Europe and Asia due to its potential clinical advantages. First, ERBT markedly enhances the quality of tumor specimens, thereby providing pathologists with a more accurate basis for evaluating the completeness of resection margins and the depth of invasion.^21^ Second, this technique employs a precise and controllable en bloc resection approach, effectively reducing the risk of perioperative complications such as obturator nerve reflex and bladder perforation.^22^ Furthermore, minimizing tumor fragmentation decreases the number of free-floating tumor cells within the bladder, theoretically reducing the likelihood of tumor reimplantation and potentially improving patients’ recurrence-free survival rates.^23^ Currently, in the realm of transurethral surgery, technologies such as holmium laser and thulium laser have been widely adopted for ERBT and have demonstrated favorable clinical outcomes. Compared with traditional plasma electroresection, laser surgery offers notable advantages, including reduced bleeding, lower complication rates, and shorter hospital stays.^24^ We posit that the key advantage of TmLER is its ability to simultaneously achieve precise cutting while preemptively coagulating and blocking tumor-supplying vessels during the resection process. In this study, patients with PUB who underwent TmLER exhibited minimal perioperative blood pressure fluctuations, and both surgical outcomes and oncological prognoses were found to be satisfactory.

Although this study provides relatively robust clinical evidence, several limitations should be acknowledged. First, given the low incidence of PUB, the sample size of the included population is relatively small, which may limit the generalizability of the findings. Thus, large-scale, multicenter clinical studies are warranted to further validate the results. Second, for PUB with a diameter less than 3 cm, transurethral surgery is recommended; for larger tumors, partial or radical cystectomy can be considered depending on specific conditions. However, due to the limited sample size, this study did not conduct a comparative analysis of the efficacy of different surgical approaches for tumors smaller than 3 cm. Therefore, additional high-quality studies are needed in the future to substantiate these recommendations.

## Conclusions

This study preliminarily confirmed the precise therapeutic efficacy of TmLER technique in treating PUB. Intraoperative blood pressure exhibited minimal fluctuation, ensuring high surgical safety, and patients demonstrated rapid postoperative recovery. It is noteworthy that for patients with PUB and smaller tumor diameter, this surgical approach represents a safe, feasible, and effective treatment alternative.

## Data Availability

The data that support the findings of this study are restricted by privacy, but might be available upon reasonable request.
